# Determination of Lactoferrin and Immunoglobulin G in Animal Milks by New Immunosensors

**DOI:** 10.3390/s90302202

**Published:** 2009-03-26

**Authors:** Luigi Campanella, Elisabetta Martini, Manuela Pintore, Mauro Tomassetti

**Affiliations:** Department of Chemistry, University of Rome “La Sapienza”, P.le A. Moro 5, 00185 Rome Italy E-Mails: luigi.campanella@uniroma1.it (L.C.); elisabettamartini@libero.it (E.M.); manuela.pintore@email.it (M.P.)

**Keywords:** Lactoferrin, immunoglobulin G, immunosensors, animal milk analysis

## Abstract

Two different immunosensors, recently developed for the determination of antibacterial proteins (lactoferrin and immunoglobulin G) in buffalo milk and in other commercial animal milks samples, were used in the present study. The aim was to propose these immunosensor methods for routine control of important diet products, such as cow and goat milks, and in particular buffalo milk. To this end we employed two different kinds of immunosensors: one for the analysis of immunoglobulin G (IgG), the other was a new amperometric immunosensor for lactoferrin analysis. Lactoferrin and IgG immunosensors were also used for the determination of lactoferrin and immunoglobulin G in buffalo milk on different days of lactation.

## 1. Introduction

Milk is known to contain various protective proteins including lactoferrin and immunoglobulin G, which can contribute to the preservation of the milk itself [1–3]. Lactoferrin is present in large quantities in mammalian secretions such as milk, tears, saliva, and seminal fluid, as well as in some white blood cells [4,5]. Lactoferrin is an iron-binding glycoprotein, which was first isolated from cow's milk and then from human milk [6–10]. Lactoferrin has many proposed biological functions, including antibacterial and anti-inflammatory activities; it also provides a defence against gastro-intestinal infections, participates in local secretory immune systems [11–13], in synergism with some immunoglobulins such as immunoglobulin G and other protective proteins, supplies an iron-binding antioxidant protein in tissues and possibly promotes growth of animal cells, such as lymphocytes and intestinal cells [14,15]. On the other hand, also immunoglobulins G are components of the immune defence mechanism by removing substances extraneous to the organism. Recent studies [16–18] have indicated that IgG in milk from regular, unimmunized dairy herds also exhibits specific antibody activity against bacteria that are pathogenic in humans. The alteration of the activity of these anti-microbial factors in cow's milk could have an impact on the shelf life of raw milk and on the development of additional health and functional foods based upon these factors. The composition of different milk samples is usually not uniform, therefore the concentrations of several milk constituents change during the lactation period and differ from one mother to the next. There are several factors that are known to influence the concentration of milk constituents in predictable ways [19,20]. These include lactation stage, breastfeeding routine, parity, age, and other maternal characteristics such as regional differences and, in some situations, season of the year and maternal diet. On the other hand, immunoglobulins (antibodies) are protective proteins that are important in the transfer of passive immunity from the mother to the child. The young of many mammalian species are born without an effective immune system, therefore the immunoglobulins and lactoferrin exhibit antimicrobial activity and protect the neonate from infection until their own immune system has developed. The increasing commercial interest in exploiting the therapeutic value of lactoferrin and IgG has stimulated the need for reliable assays for their determination at the endogenous level in milk [21–23]. This study is aimed at testing immunosensor methods for the measurement of antibacterial proteins (lactoferrin and immunoglobulin G) in buffalo milk and in commercial cow and goat milks, with a view to proposing these immunosensor methods for routine control of milk. To this end we employed two kinds of immunosensors: one recently developed for the quantification of lactoferrin, [24] and another selective one for the analysis of immunoglobulin G, already described in a previous paper [25]. Both were used for the measurement of lactoferrin and immunoglobulin G in different animal milk samples. In addition, the antioxidant capacity of buffalo milk samples was also measured with a superoxide dismutase (SOD) biosensor, developed in our laboratory [26–28]. Finally lactoferrin and immunoglobulin G concentration trends and those of antioxidant capacity were compared as a function of the buffalo’s lactation days and are briefly discussed.

## 2. Experimental Section

### 2.1 Apparatus

The amperometric measurements were carried out in a 5 mL thermostated glass cell kept under constant stirring. The amperometric measurements for the oxygen were performed using an oximeter (Amel model. 360, Milan, Italy), connected to a recorder (Amel mod. 868) and a Clark electrode supplied by Amel (mod. 332). For the amperometric H_2_O_2_ measurements an Amel mod. 551 potentiostat was used, coupled with an amperometric hydrogen peroxide electrode by Universal Sensor Inc. (New Orleans, LA, U.S.A.), Mod. 4006, and connected to an Amel mod. 868 analog recorder. For the SOD biosensor measurements an Amel mod. 551 potentiostat was used coupled with a mod. 4000^−1^ electrode supplied by Universal Sensor Inc. and connected to an Amel mod. 631 differential electrometer and an Amel mod. 868 analog recorder.

### 2.2. Materials

Ny+ Immobilon Affinity membrane, a positively charged nylon membrane with polyester reinforcement optimized for reliable and reproducible transfer, immobilization, hybridization, and subsequent reprobing, porosity 0.65 μm, was from Millipore Corporation (catalog number INYC08550; New York, USA). Polyclonal anti-lactoferrin produced in rabbit (catalogue number L-3262), lactoferrin from bovine milk (catalogue number L-9507), and the biotinylation kit, supplied by Sigma Immunochemicals (St. Louis, MO, USA), composed of: biotinylation reagent (BAC-SulfoNHS i.e. biotinamido hexanoic acid 3-sulfo-*N*-hydroxysuccinimide ester), 5 M sodium chloride solution, micro-spin Column (2 mL, practically consisting of a small empty cylindrical vessel pre-packaged with Sephadex G-50), 0.1 M Sodium Phosphate Buffer pH 7.2, 0.01 M Phosphate Buffer Saline (PBS) pH 7.4 (reconstituted with 1 liter of deionized water to give 0.01 M Phosphate Buffer, 0.138 M NaCl, 2.7 mM KCl, pH 7.4); Extravidin® peroxidase (containing 0.2 mL of extravidin Peroxidase conjugate at 2.0 mg/mL, supplied with 0.01% thimerosal), xanthine (2,6-dehydroxypurine) sodium salt, ethylenediamine tetracetic acid (EDTA), superoxide dismutase 4980 U/mg, albumin (from bovine serum) (BSA), TRIS (hydroxymethylaminomethane), TWEEN^®^ 20, dialysis membrane (art. D-9777), phenol, formic acid, cellulose triacetate (TAC), anti-bovine IgG (whole molecule)−alkaline phosphatase antibody produced in rabbit (catalog number A0705), anti-goat IgG (whole molecule)–alkaline phosphatase antibody produced in rabbit (catalog number A4187), bovine IgG (Sigma I-5506), goat IgG (catalog number I5256) were obtained from Sigma-Aldrich (St. Louis, MO, USA); xanthine oxidase 0.39 U/mg, kappa-carrageneen, tyrosinase (EC. 1.14.18.1) extract from mushroom 3216 U/mg were supplied by Fluka (AG, Buchs, Switzerland); magnesium chloride, potassium phosphate monobasic, potassium phosphate bibasic and all other solvents or reagents of the highest purity were from Carlo Erba (Milan, Italy).

### 2.3. Sample analyzed

Nine raw buffalo milk samples were drawn from the animal on different days during a normal lactation period on a dairy farm in the Pontine area (Lazio, Italy). A veterinarian declared the buffalo healthy. Two different samples of commercial fresh milk (i.e. goat and cow milk) and two commercial yoghurts (containing pineapple and wild berries, respectively) were also analysed. All samples were purchased from a local drugstore.

## 3. Methods

### 3.1. Lactoferrin biotinylation and extravidin-peroxidase conjugation

**Figure 1.**
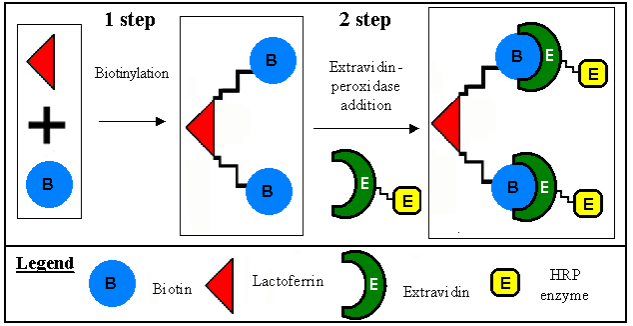
Biotinylation and conjugation of the lactoferrin.

The avidin-biotin peroxidase technique is based on the use of a biotinylated antibody and an avidin horseradish peroxidase conjugate as part of the labelling system. The technique exploits the high affinity binding of biotin to avidin. The BiotioTag kit is specially designed for the small scale labelling of antibodies using biotinamido hexanoic acid 3-sulfo-*N*-hydroxysuccinimide ester (BAC-SulfoNHS) as the labelling reagent. This reagent is particularly useful when mild reaction conditions are required for the biotinylation of sensitive biomolecules such as antibodies, enzyme and surface proteins. Following the labelling reaction, the biotinylated protein is separated from unreacted or hydrolyzed reagent by a fast gel-filtration step using G-50 microspin columns. BAC-SulfoNHS reacts with free amino groups of proteins to form stable amide bonds. Extravidin binds to biotin with a high affinity (Ka = 10^15^ M) and specificity. High affinity for biotin alleviates non-specific binding interactions commonly associated with the strongly basic avidin protein [29–31]. The use of the extended spacer arm greatly improves the interaction between extravidin and the biotinylated macromolecule thus overcoming steric hindrance present at the biotin binding sites of extravidin [32]. The full procedure is illustrated in Figure 1 for the antigen biotinylation and extravidin-peroxidase conjugation.

Briefly: 0.1 mL of 1.0 mg/mL lactoferrin solution in sodium phosphate buffer, (pH 7.2; 0.1 M) was prepared. Separately a 5 mg/mL BAC-SulfoNHS solution was also prepared, by dissolving 5 mg of biotinamido hexanoic acid 3-sulfo-*N*-hydroxysuccinimide ester in 30 μL DMSO and adding sodium phosphate buffer (pH 7.2; 0.1 M) to a final volume of 1 mL. Immediately 10 μL of BAC-SulfoNHS solution were added to the lactoferrin solution with gentle stirring and the mixture incubated under stirring for 30 minutes at room temperature. Then the resin was re-suspended in the column by vortexing, the column was equilibrated with 0.2 mL of PBS, (pH 7.40; 0.01 M), (this buffer was required both as an equilibration buffer of the microspin G-50 column and for the elution of the labelled protein from the column). The biotinylation reaction mixture was applied to the top-center of the resin and the column was centrifuged for 5 minutes at 3,000 rpm. The purified sample was collected at the bottom in an Eppendorf test tube. This step was repeated twice more and a total of three fractions were collected. Lastly the extravidin peroxidase solution (20 μL, 2.0 mg/mL), diluted 1:100 in PBS containing 1% BSA, was added to the collected sample and incubated with it for 1 hour at room temperature, and lastly rinsed gently with PBS, (pH 7.4; 0.01 M), to remove the extravidin peroxidase solution in excess.

### 3.2. Anti-lactoferrin immobilization on Immobilon membrane

The Immobilon Ny+ Membrane was cut into approximately 1 cm^2^ surface area disks and 100 μL of a 1.0 mg/mL anti-lactoferrin was directly deposited on the membrane surface. The membrane was then dried at room temperature for about 24 h and stored at 4° C.

### 3.3. Immunosensor assembly

**Figure 2.**
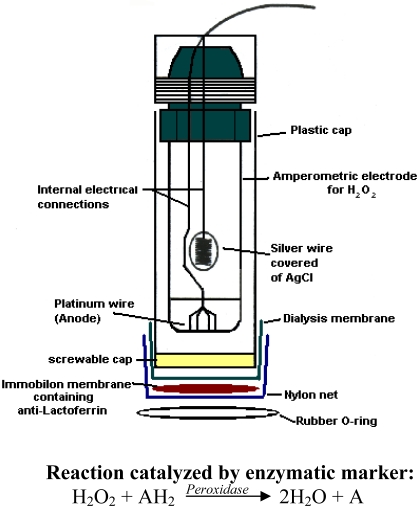
Amperometric immunosensor for lactoferrin determination using hydrogen peroxide electrode as transducer.

The transducer consisted of an amperometric electrode for H_2_O_2_ determination, with a Pt anode and an Ag/AgCl/Cl^−^ cathode, provided with a plastic cap filled with 0.1 M KCl solution and screwed onto the body of the electrode, at the lower end of which a dialysis membrane was positioned. The Immobilon membrane with the immobilized anti-lactoferrin overlapped the dialysis membrane. Finally, a nylon net overlapped the latter membrane. The two membranes and the net were secured by a rubber O-ring to the plastic cap of the electrode as shown in Figure 2.

### 3.4. Determination of lactoferrin by immunosensor

**Figure 3.**
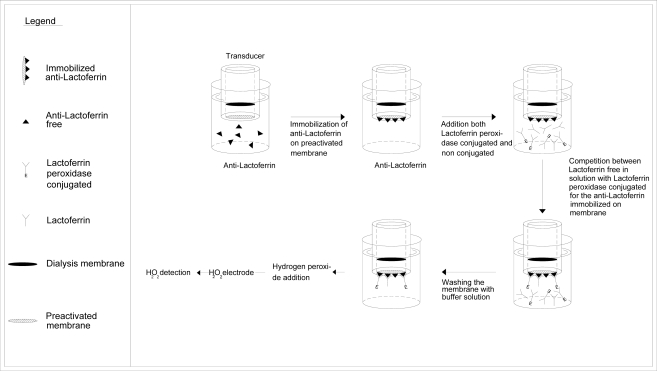
Determination of lactoferrin by immunosensor, Test geometry: competition between lactoferrin biotin-avidin-peroxidase conjugated and lactoferrin, both free in solution for Anti-lactoferrin immobilized in membrane.

Competition procedure: competition between lactoferrin biotin-avidin-peroxidase conjugated and non conjugated lactoferrin, both free in solution, for anti-lactoferrin immobilized in membrane. To this end, the Immobilon membrane, on which the anti-lactoferrin was immobilized, was fixed to the head of the amperometric electrode for hydrogen peroxide as described in Section 3.3. Before measurement, the immunosensor was dipped into a Tris-HCl buffer solution, (pH 8.0; 0.1 M), containing 0.05 % Tween^®^-20 by weight and 2.5% BSA by weight (bovine albumin was used to minimize non specific absorption on the membrane). The lactoferrin sample to be determined was added in 5 mL of Tris-HCl buffer solution (pH 8.0; 0.1 M) contained in the measurement cell, together with a fixed supply of lactoferrin biotin-avidin-peroxidase conjugated, i.e. 20 μL (2.0 mg/mL) of conjugated lactoferrin. The peroxidase-conjugated lactoferrin was allowed to compete with the non-conjugated lactoferrin, both free in solution, in binding with the anti-lactoferrin immobilized on the Immobilon membrane. After washing with the same buffer solution to remove all the unbound lactoferrin, the specific substrate of the enzyme, i.e. 20 μL of H_2_O_2_ solution 1% v/v, was added to the renewed buffer solution in which the immunosensor was dipped, under stirring. The measured signal (as nA) of the transducer correlated directly with the lactoferrin concentration to be measured. In this case, the higher the concentration of non conjugated lactoferrin free in solution, the stronger the signal produced by the hydrogen peroxide. Indeed, the lower the conjugated lactoferrin bound to the antibody immobilized on Immobilon membrane, the lower the H_2_O_2_ consumed in the enzymatic reaction, and therefore the higher the signal of the H_2_O_2_ oxidized at the amperometric electrode. The sequence for measuring the lactoferrin by this procedure is schematized in Figure 3.

**Figure 4.**
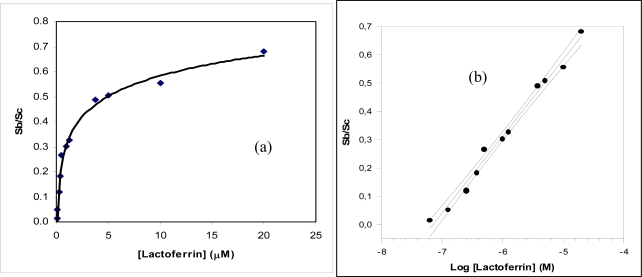
(a) Behaviour of the lactoferrin immunosensor response as a function of increasing lactoferrin concentration, using Immobilon membrane and an amperometric electrode for H_2_O_2_ as transducer; (b) corresponding calibration curve and confidence interval for the lactoferrin determination, (Sc = sample signal/nA; Sb = blank signal/nA).

The lactoferrin immunosensor response using this procedure is shown in Figure 4(a), while a calibration curve, shown in Figure 4(b), was constructed by the same data of as shown in Figure 4(a) and employed to determine the unknown concentration of lactoferrin contained in the sample.

### 3.5. IgG immobilization on Immobilon membrane

The Immobilon Ny+ Membrane was cut into disks of approximately 1 cm^2^ surface area and 25.0 μL of a 50 mg/mL Immunoglobulin G solution was directly deposited on the surface of each disk. The membrane was then dried at room temperature for about 24 h and stored at 4° C before being used.

### 3.6. Construction of immunosensor for IgG measurements

**Figure 5.**
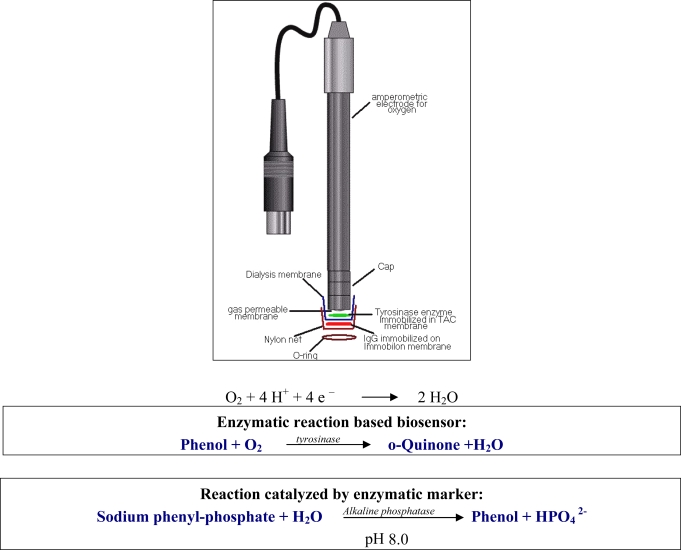
Immunosensor for IgG determination.

The transducer was a tyrosinase enzyme biosensor, fabricated using an oxygen amperometric electrode coupled to the tyrosinase enzyme (Figure 5), immobilized in TAC membrane [25] and based on the following enzymatic reaction:

$$\text{Phenol}+O_{2}\;\;\;\;\;\underset{\to }{\text{tyrosinase}}\;\text{o-Quinone}+H_{2}O$$

The immunosensor assembly was described in a previous paper [21] and is schematized in Figure 5.

### 3.7. Determination of IgG by new immunosensor

Standards of IgG free in solution at different concentrations, or IgG contained in samples to be determined was allowed to compete with the same antigen but immobilized on the Immobilon membrane overlapping the head of the amperometric electrode for oxygen, in order to produce the antibody reaction with a fixed supply of antibody, free in solution and labelled with alkaline-phosphatase enzyme.

**Figure 6.**
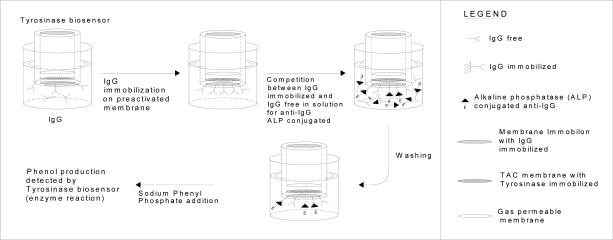
Determination of antigen (IgG) by new immunosensor using tyrosinase enzyme electrode as a transducer. Test geometry: competition for anti-IgG alkaline phosphatase conjugated between IgG immobilized on membrane and IgG free in solution.

In practice, before measurement, the immunosensor was immersed in 5 mL of 0.1 M Tris-HCl buffer solution containing 0.05 % Tween^®^-20 and 2.5 % by weight BSA (in order to minimize non specific absorption on the membranes); then the Tris-HCl buffer solution, 0.1 M, pH 8.0 was renewed in the cell in which the IgG to be determined, together with a fixed concentration, i.e. of 20 L (2 mg/mL) in 5 mL of Tris buffer, the enzyme-labelled anti-IgG (that is anti-IgG-alkaline-phosphatase conjugate), was allowed to incubate at 25° C for 1 h. The free in solution antigen (IgG) competes with the IgG immobilized on the membrane of the immunosensor dipping into the same solution in binding the labelled anti-IgG. On adding the enzyme substrate (phenyl-phosphate) to the renewed buffer solution, after washing with the same buffer to remove all the unlabelled anti-IgG not bound to the IgG, the recorded signal was correlated with the quantity of labelled immunocomplex formed on the surface of the membrane and inversely correlated with IgG concentration to be measured. The calibration curve obtained by plotting the current signal versus the final log IgG concentration was then used to determine the concentration of the unknown anti-IgG. In practice the sequence of events occurring during the IgG assay is outlined in Figure 6.

**Figure 7.**
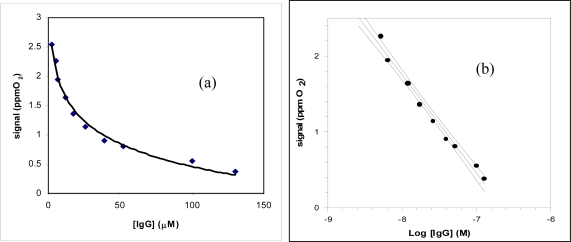
(a) Behaviour of the IgG immunosensor response as a function of increasing IgG concentration using Immobilon membrane and tyrosinase biosensor as a transducer; (b) corresponding calibration curve and confidence interval for IgG determination.

The response of the immunosensor with increasing IgG concentration is shown in Figure 7 (a). The calibration curve (see Figure 7(b)) obtained by plotting the current signal versus the final log IgG concentration was then used to determine the concentration of the unknown IgG.

### 3.8. Determination of lactoferrin and immunoglobulin G in different milk samples

For the purpose of analyzing lactoferrin in all the commercial (cow and goat) milk samples and in samples of raw buffalo milk, as well as two yoghurt samples, 2.5 mL of sample was added directly to the measuring cell containing 2.5 mL of 0.1 M phosphate buffer solution (pH 7.2) and 0.5 M sodium chloride. For the purpose of IgG determination 200 μL respectively of commercial (goat or cow) milk, or else both yoghurt samples were added to the measuring cell containing 5 mL of 0.1 M phosphate buffer solution (pH 7.2) and 0.5 M sodium chloride. For IgG determination in buffalo milk it was sufficient simply to add 100 μL only of the sample. Lactoferrin concentration in the milk samples withdrawn in this way, was then measured using the lactoferrin immunosensor described in Section 3.3 and the competitive immunoassay procedure described in Section 3.4. Likewise the immunoglobulin G concentration was measured in the same samples, withdrawn in the same way, by the immunosensor for the immunoglobulin G, described in detail in a previous paper [25] and using the competitive immunoassay procedure described in the Section 3.7.

### 3.9. Determination of antioxidant capacity by SOD biosensor

The relative antioxidant capacity of the nine buffalo milks at different lactation times was determined by the SOD biosensor method, as optimized in our laboratory [26–28]. Briefly the antioxidant activity of the milk samples was checked using the superoxide dismutase (SOD) electrochemical biosensor, measuring the superoxide radical variation related to the antioxidant capacity of the sample. The biosensor used to determine the superoxide radical was obtained by coupling a transducer (an amperometric hydrogen peroxide electrode) with the superoxide dismutase enzyme, immobilized in kappa-carrageenan gel [26–28]. The superoxide radical (O_2_^.−^) is produced in aqueous solution by xanthine, which is converted to uric acid during the oxidative reaction catalyzed by the xanthine oxidase enzyme free in solution. The disproportion reaction of the O_2_^.−^ radical in the presence of the superoxide dismutase enzyme, immobilized in gel membrane overlapping the electrode, produces oxygen and hydrogen peroxide. The latter is oxidized at the platinum anode, producing a current signal which decreases in the presence of a scavenging species able to react with the O_2_^.−^ radical. This decrease allows the measurement of the relative antioxidant capacity (RAC) [26–28].

## 4. Results and Discussion

Figure 4(a) shows the behaviour of the response of the immunosensor for lactoferrin determination obtained using the competitive procedure and the amperometric sensor for hydrogen peroxide as transducer, while Figure 7(a) shows an analogous response of the immunosensor for the immunoglobulin G determination, equipped with a Clark electrode. The corresponding calibration straight lines obtained from the same data are shown in Figures 4(b) and 7(b), respectively. The main results for lactoferrin and IgG determination as in regards to analytical characterization and the respective equations of calibration straight lines reported in Figures 4(b) and 7(b) are summarized in Table 1 and show that the lower detection limit (LOD) for lactoferrin is of the order of 35 nM, while in the case of IgG the LOD is of the order of 1.3 nM; the RSD% (percent relative standard deviation) are sufficiently low for both the immunosensors. Lastly the linear range is about two decades for IgG and about two decades and a half for lactoferrin, while the recovery data obtained applying the standard addition method on standard solution are certainly good.

Table 2 summarizes the lactoferrin and IgG concentrations obtained respectively using the two immunosensors described in the preceding sections for different animal (cow, buffalo and goat) milks as well as for two dairy milk products (yoghurt). It can be seen how lactoferrin concentration is much higher in raw buffalo milk and raw cow milk than in fresh commercial cow and goat milk samples.

In the case of IgG, a concentration of the same order of magnitude is found both in buffalo milk and in cow milk. Conversely, in the goat milk sample, the observed value is about one third of that of the other two. Lastly, the two yoghurt samples give concentrations at least one order of magnitude lower than the milk samples. In both cases (i.e. IgG and lactoferrin) measurement repeatability was found to be satisfactory (RSD % ≤ 5.5). The possibility of interference in the lactoferrin and IgG analysis was also evaluated by performing the standard addition analysis of several milk samples, at different dilutions (Tables 3 and 4). Results show that the recovery of added lactoferrin and IgG was always close to 100% in any case independent of the dilution level, which confirmed the absence of any significant matrix interferent. Indeed, the experimental differences with respect to the 100% theoretical recovery values are purely random.

This data also provides additional evidence that the analytical signal is not compromised if any non-specific interactions occur between other species possibly contained in real samples and the membrane surface. On the other hand, the addition of 0.5 M sodium chloride to the phosphate buffer solution used for the measurement, by increasing the ionic strength of the solution, reduces the possible interaction of the other milk proteins with the hydrophilic exchanger of the membrane surface.

Lastly if we compare the principal analytical data obtained for the sensor for the lactoferrin or that for the IgG with those found by applying other analytical methods that are similar or else very different (see Table 5), it is observed that the LOD value for the IgG is one of the best, while for the lactoferrin it is of the same order of magnitude as three of the six tabulated methods, better than a fourth and worse than only the remaining two methods. Furthermore, the linearity range in the case of lactoferrin is larger than that of all the other tabulated methods [22,33–43].

The above results thus justify the fact that, in this work, the lactoferrin and IgG assays by immunosensors were applied to buffalo milk samples after simple dissolution of the milk sample in phosphate buffer, thus obtaining a final dilution level of 1:50–1:100 v/v for lactoferrin determination and 1:3,000–1:1,000 v/v for IgG determination. It is possible to use this simplified protocol as the LOD of both immunosensors is sufficiently low. In addition it is evident as the reduction of the number of manipulations using immunosensors, compared with other methods [38,39,44,45] in which these pretreatments are instead necessary, averts the danger of recovery losses. For instance, as lactoferrin is associated with casein, the removal of the latter leads to the introduction of filtration and centrifugation protocols, which must be utilized when alternative analytical techniques are used [38,46]. On the other hand, it is important that a good lactoferrin measurement method allows measures to be carried out also at very low concentrations, at least of the order of 20 mg/L or lower, since it is reported in the literature [21–23,47–49] that considerable variations of lactoferrin concentration were observed (high or very low) in the different milk samples.

As soon as it was found possible to perform correct measures on different real milk samples using the immunosensors described, it became possible to observe the variations in lactoferrin and IgG concentrations in the buffalo milk during the animal’s lactation period. In the nine analyzed samples of buffalo milk, each referring to a different lactation day, the lactoferrin and the immunoglobulin G concentrations were determined using the two immunosensors described herein. Lastly the relative antioxidant capacity (RAC) was also checked, with a SOD biosensor method, as briefly described in section 3.9 [26–28].

Buffalo lactation can be divided into four phases that differ in the composition and volume of milk produced: colostral, transitional, mature, and involutional [50,51]. Colostrum is secreted up to five days after delivery, transitional milk up to the end of the second week, mature milk during the remaining full lactation days, and involutional milk from the end lactation of the lactation period on. Of course these definitions are relatively arbitrary, as these phases vary from one mother cow to another, while the milk composition does not change abruptly; however milk volume is low during the colostral phase, rising slowly during the first week to the higher levels of established lactation [50]. Colostrum is richer in secretory IgA and IgG, lactoferrin, vitamin A, and sodium, compared with mature milk, but has relatively low concentrations of fat, lactose, and vitamin B1 [37,52,53]. Lactoferrin is one of the proteins that occur naturally in buffalo milk at an average concentration of about 200 mg/L, but in the colostrum, the lactoferrin content can be as high as 500 to 1,000 mg/L [50]. Mature milk composition also changes during the lactation phase, although not as markedly as in the early weeks. The lactoferrin content of the analysed buffalo milk samples in almost all cases lies within the linear interval of the immunosensor method described herein (about 5.0–800 mg/L). The nine buffalo milk samples analyzed in the present research may be considered to belong to the “mature period of lactation” after about 40th day of lactation.

In seven of the nine analyzed samples drawn after about one month lactation the lactoferrin concentration found, reported in Table 5, ranges from about 53 mg/L to 500 mg/L and decreases with the number of lactation days, while in the other two analyzed samples, the lactoferrin concentration ranges from about 660 mg/L to 800 mg/L. The Immunoglobulin G concentration over the same period ranges from about 170 mg/L to 1,075 mg/L and usually increases with the number of lactation days, although in a way, not at all, monotonous.

**Figure 8.**
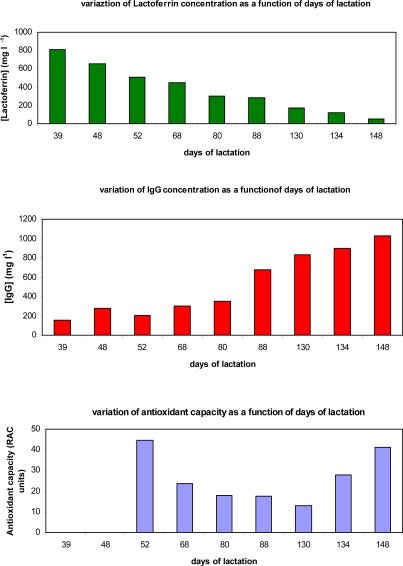
Trends in: (a) lactoferrin concentration; (b) IgG concentration; (c) Antioxidant capacity; in buffalo milk samples as a function of increasing days of lactation.

This increase may seem not be justified on the basis of the observations of several authors [54–57], according to whom IgG concentration in animal milk decreases rapidly during the colostrum period and, during the mature phase, in any case remains lower than during the colostrum phase [58, 59]. In our case, however, all the samples belonged to the mature lactation period, during which IgG concentration may either increase or decrease depending on different factors, such as the time of year, the animal’s physiological status, feeding [59,60]; for example, the concentration increases when inflammatory conditions are present, such as mastitis [54,57,58,61]. Such a circumstance could for instance account for the IgG values found by us between day 39 and day 148 of lactation. It is in any case singular to observe how, over the same time interval, the trends of lactoferrin and immunoglobulin G levels, shown in Figure 8, seem to point to the existence of a degree of quasi inverse correlation of the concentration of the two protein types with increasing lactation time. It might be postulated that a kind of “compensation” occurs that tends to maintain a sufficiently high level of antimicrobial protection provided by the foodstuff. However, at the present state of the research, this can only be considered a working hypothesis; that the IgG trend is to be considered much more complex may be inferred from the fact that, in two buffalo milk samples taken after day 148 of lactation (that is, on day 155 and day 160), IgG concentration was already found to be lower than that found at the end of the time interval shown in figure 8. The antioxidant capacity trend, shown in figure 8, is certainly more difficult to interpret. It can only reasonably be hypothesized that the initially higher value may be related also to the high lactoferrin concentration in view of its well-known antioxidant capacity [61–67], although this may be considered no more than a mere hypothesis, the verification of which is even more necessary with respect to the hypothesis expressed in the preceding case as it is a known fact that milk contains higher concentrations of other antioxidant species which certainly play a more important role [67–70].

## 5. Conclusions

The increasing commercial interest in exploiting the therapeutic value of lactoferrin and IgG in milk has stimulated the need for reliable assays for their determination at the endogenous level in buffalo milk samples and in other cow or goat milk samples. In this context two immunosensors allowing a direct measurement of lactoferrin and immunoglobulins G in milk products were described and assessed from the analytical standpoint in their application to various animal milk samples and derived products. The sensitivity, linear range and LOD of two immunosensors were determined and proved satisfactory for the animal milk analysis. Since the bioactive proteins contained in bovine milk, such as lactoferrin and IgG, will be increasingly exploited in the expanding international trade of milk products [51,69], in the absence of any currently accepted reference method, the two proposed immunosensor methods may fulfil this need for lactoferrin and IgG determination in animal milks. The IgG and lactoferrin level variation in buffalo milk during lactation was also successfully studied in the present research using new immunosensors for lactoferrin and IgG analysis. In addition, an enzymatic SOD-biosensor, applied to detect the antioxidant capacity of buffalo milk during lactation, displayed a significant variation in antioxidant capacity with the passing of time. However a considerable amount of experimental research in different directions, particularly as far as the antioxidant capacity of vitamins contained in the milk is concerned [71,72], i.e. the evaluation of vitamin B (as biotin, niacin and folic acid), vitamins C, D and E, moreover both the carotene and ß-lactoglobulin variation during lactation, will be required to provide a correct interpretation of the antioxidant capacity trend found in the present research.

## Figures and Tables

**Table 1.** Analytical characterization of two immunosensor methods for lactoferrin determination and for IgG determination using competitive procedures.

**Methods**	**Determination of lactoferrin by means of immunosensor. Test geometry: competition between lactoferrin biotin-avidin-peroxidase conjugated and lactoferrin free in solution for anti-lactoferrin immobilized in membrane^A^**	**Determination IgG by means of new immunosensor that uses as transducer a tyrosinase enzyme electrode. Test geometry: competition for the free in solution anti-IgG conjugated with the alkaline phosphatase, between the IgG immobilized on the membrane and IgG free in solution^B^**
Regression equation (Y = a.u., X = μM) confidence level (1- α) = 0.95;	Y = 0.27 (±0.04) log X + 0.31 (±0.02) (n – ν) = 9 ; (t = 2.26)	Y = −1.01 (±0.26) log X + 1.7 (±0.04) (n – ν) = 7 ; (t = 2.36)
Linear range (μM)	(0.7 – 100) × 10^−1^	(2.6 – 130) × 10^−3^
Correlation coefficient	0.9891	0.9885
Pooled SD%	≤5.8	≤5.7
Low detection limit (LOD) (μM)	3.5 × 10^−2^	1.3 × 10^−3^
Recovery of standard solution (% recovery values found in the linear range)	(99.2–100.3)%	(99.4–100.8)%
Repeatability of the measurement as relative standard deviation (RSD%)	≤5.5	≤5.4
Instrumental response time (min)	5	10

A Operating conditions: Buffer solution: Tris (0.1 M), pH 8.0; Incubation temperature 25 °C; Incubation time: 60 min. Membrane employed: Immobilon membrane.

B Operating conditions: Buffer solution: Tris (0.1 M), pH 8.0; Incubation temperature 25 °C; Incubation time: 60 min. Membrane employed: Immobilon membrane.

**Table 2.** Determination by immunobiosensor of lactoferrin and IgG concentration values in cow, goat and buffalo milk and in two dairy milk products (yoghurt). Values expressed both as mg/L and as μM.

**Milk or dairy matrix**	**Found lactoferrin concentration (mg/L) n = 5; RSD% ≤ 5.5**	**Found lactoferrin concentration (μM) n = 5; RSD% ≤ 5.5**	**Found IgG concentration (mg/L) n = 5; RSD% ≤ 5.4**	**Found IgG concentration (μM) n = 5; RSD% ≤ 5.4**
Raw Cow Milk	182.4	2.28	772.5	5.15
Cow Milk (UHT conservation)	18.3	0.23	620.0	4.13
Goat Milk (Partially skimmed)	17.5	0.22	220.0	1.46
Raw “Buffalo” milk	232.0	2.90	675.0	4.50
“Fruit of wood” Yogurth	8.0	0.10	39.1	0.26
“Pineapple” Yogurth	7.8	0.09	42.5	0.28

**Table 3.** Recovery tests of added lactoferrin in milk and in several dairy milk products using the standard addition method.

**Milk Matrix**	**Found lactoferrin concentration (μM) (n=5); RSD% ≤ 5.5**	**Added lactoferrin concentration (μM)**	**Experimental lactoferrin concentration (μM) (n=5); RSD% ≤ 5.5**	**Recovery % lactoferrin concentration in milk matrix**
Buffalo milk (Diluted 1:100)	2.58 × 10^−2^	1.0 × 10^−2^	3.94 × 10^−2^	100.6
Buffalo milk (Diluted 1:50)	5.96 × 10^−2^	2.0 × 10^−2^	7.83 × 10^−2^	98.4
Cow milk (UHT conservation) (Diluted 1:10)	2.3 × 10^−2^	1.0 × 10^−2^	3.7 × 10^−2^	112.1
Cow milk (UHT conservation) (Diluted 1:5)	4.5 × 10^−2^	5.0 × 10^−2^	9.2 × 10^−2^	96.8

**Table 4.** Recovery tests of added IgG in cow and buffalo milk using the standard addition method.

**Milk Matrix**	**Found IgG concentration (μM) (n = 5); RSD% ≤ 5.4**	**Added Ig G concentration (μM)**	**Experimental IgG concentration (μM) (n = 5); RSD% ≤ 5.4**	**Recovery % IgG concentration in milk matrix**
Buffalo milk (Diluted 1:1,000)	6.15 × 10^−3^	1.0 × 10^−3^	7.27 × 10^−3^	101.7
Buffalo milk (Diluted 1:500)	1.38 × 10^−3^	2.0 × 10^−3^	3.32 × 10^−3^	98.2
Cow milk (UHT conservation) (Diluted 1:2,000)	2.2 × 10^−3^	1.0 × 10^−3^	2.9 × 10^−3^	90.6
Cow milk (UHT conservation) (Diluted 1:3,000)	1.6 × 10^−3^	0.5 × 10^−3^	2.4 × 10^−3^	114.3

**Table 5.** Comparison of analytical data found by several analytical methods for IgG or Lactoferrin analysis.

**IMMUNOGLOBULIN G**				**LACTOFERRIN**			
References	Method	Linear range (μg/mL)	LOD (μg/mL)	References	Method	Linear range (μg/mL)	LOD (μg/mL)
[this work]	immunosensor	0.4 – 20	0.2	[this work]	immunosensor	5.6 – 800	2.8
[33]	ELISA	1–100	0.3	[39]	ELISA	0.05 – 10	0.01
[34]	SPR–Immunoa ssay	0.015–10	80	[40]	ELISA	3.12 – 200	1.0
[35]	Immunonephelometry	50–200	30	[22]	Optical biosensor	0.004 – 1.0	0.002
[36]	Immunonephelometry	0.05–0.8	0.008	[41]	RP-HPLC method	0.2 – 30	0.2
[37]	Affinity LC	10–150	0.5	[42]	Spectrometric method	10–100	1
[38]	RID	300–1400	120	[43]	Immuno-affinity Chromatography	20–200	12
